# Supplementary material for: Social-structural barriers to primary care among sex workers: findings from a community-based cohort in Vancouver, Canada (2014–2021)
Source: BMC Health Serv Res. 2025 Jan 24;25:134. doi: 10.1186/s12913-025-12275-x (PMC11760670; doi:10.1186/s12913-025-12275-x)
Supplement: Supplementary file 1 — Supplementary Material 1. [file 12913_2025_12275_MOESM1_ESM.docx]

| Study variables derived from An Evaluation of Sex Workers Health Access Questionnaire, 2014 to 2021 | | | |
| --- | --- | --- | --- |
| Variable name | Question/s* | Questionnaire Responses* | Responses for analysis |
| Outcome | | | |
| Primary care use | In the last six-months, have you seen a family doctor? | - Yes   vs   - No | - Yes   vs   - No |
| Explanatory variables | | | |
| Intimate partner violence | Have you ever experienced any of the following by your  male intimate partners (boyfriends, spouses)? In the last 6  months?  (WHO Intimate Partner Violence Scale)  Moderate Physical IPV  1) Slapped you or thrown something at you that  could hurt you?  2) Pushed or shoved you?  Severe Physical IPV  3) Hit you with a fist or with something else that  could hurt?  4) Kicked you, dragged you, or beaten you up?  5) Choked or burnt you on purpose?  6) Threatened to use or actually used a gun, knife, or other weapon against you?  Sexual IPV  8) Physically forced you to have sexual intercourse  when you did not want to?  9) Had sexual intercourse when you did not want to  because you were afraid?  10) Forced to do something sexual that you found  degrading or humiliating?”  Emotional IPV  11) Insulted or made you to feel bad about yourself?  12) Belittled you or humiliated you in front of other  people?  13) Done things to scare or intimidate you on purpose  (e.g. by the way he looked at you, by yelling or  smashing things)?  14) Threatened to hurt someone you care about? | For each question:   - Yes   vs   - No | - Yes (‘Last 6mo’ to any options under ‘Moderate Physical IPV’, ‘Severe Physical IPV’, ‘Sexual IPV’)   vs   - No (None of the above or no intimate male partner(s) in the L6M) and/or no intimate male partner(s) in the L6M’ |
| Violence when doing sex work | In the last 6 months, have you experienced any of the  following bad dates/ violence by clients?  ▢ Verbal harassment/ threatened  ▢ Ripped off  Abducted/kidnapped  ▢ Forced to have sex without a condom  ▢ Broke condom on purpose  ▢ Removed condom on purpose (“stealthing”)  ▢ Raped (forced sex against will)  ▢ Sexual assault (any unwanted sexual advances/  violence, including touching, grabbing, etc.)  ▢ Strangled  Physically assaulted/ beaten  Locked/ trapped in car  ▢ Thrown out of moving car  ▢ Assaulted with a weapon  ▢ Stranded/ dropped off in a location against your  Will  ▢ Drugged  Trapped in room/hotel/housing,etc.  ▢ Other (specify:_______) | For each option:   - Yes   vs   - No   OR   - N/A (never had any violent/ threatening clients) | - Yes (‘Yes’ to any)   vs   - No (None to all and/or NA) |
| Limited English fluency | How comfortable do you currently feel  1. Speaking English  2. Reading English  3. Writing English | For each question:   - Very Comfortable   vs   - Somewhat comfortable   vs   - Not very Comfortable   vs   - Uncomfortable   vs   - Very Uncomfortable | - Yes (‘Not very comfortable’, ‘Uncomfortable’, ‘Very uncomfortable’ to ‘Speaking English’)   Vs   - No (‘Very comfortable’, ‘Somewhat comfortable’ to ‘Speaking English’) |
| Precarious immigration status | Were you born in Canada? | - Yes   vs   - No | - Yes   vs   - No |
| Health care stigma | In the last six months?  1. Have you been denied health services (or someone kept you from receiving health services because you sell sex?)  2. Have you felt that you were not treated well in a health centre because you sell sex?  3. Have you heard healthcare providers gossiping about you because you sell sex? | For each question:   - Yes   vs   - No | - Yes (if responded yes to any)   vs   - No (if responded no to all) |
| Unstably housed | In which of the following types of places have you slept overnight in the last 6 months?  ▢ Apartment/ house alone  ▢ Apartment/house with roommates  ▢ Apartment/house shared with intimate  partner  ▢ Apartment/house shared with family  (intimate partner and children or just children)  ▢ Staying with parents  Staying with family/relative (other than  parents)  ▢ Supportive housing  **Other**  ▢ Transition House  Shelter/ hostel (specify:_____________)  ▢ Hotel (hourly/ nightly rental)  ▢ Sex work homestay  ▢ Treatment/ recovery house  (specify:_________________________)  ▢ Couch surfing  ▢ Staying with friend  ▢ Boyfriend’s place  ▢ Girlfriend’s place  ▢ Ex-partner’s place  ▢ Client’s place  ▢ Psychiatric facility  ▢ Hospital  ▢ In a vehicle  ▢ On the street (alley/park)  ▢ Squat  ▢ Jail  ▢ WISH | For each option   - Any   And/or   - Current | - Yes (to any of ‘SRO hotel’, ‘Staying with parents’, ‘Staying with family/relative’, any ‘Supportive housing’, any ‘Other’)   vs   - No (ONLY answered Any (6mo)’ to ‘Apartment/house alone’, ‘Apartment/house with roommates’, ‘Apartment/house shared with intimate partner’, ‘Apartment/house shared with family') |
| Incarceration | In the last 6 months, have you been in detention, prison or jail overnight or longer for any reason at all? | - Yes   vs   - No | - Yes   vs   - No |
| Police harassment when doing sex work | Have you ever experienced any of the following encounters with police? In the last 6 months?  ▢ Arrested/ put in jail  ▢ Told to move on  ▢ Police inspection  ▢ Police raid (indoor space)  ▢ Threatened with arrest/ detainment/ fine  ▢ Searched (without arrest)  ▢ Followed  ▢ Picked up and driven elsewhere to work  ▢ Verbally harassed  ▢ Stopped, name run, ID checked  ▢ Parked nearby/drove by repeatedly  ▢ Detained (delayed/ held against will without arrest)  ▢ Physically assaulted  ▢ Drugs/ drug use equipment taken (without arrest)  ▢ Condoms taken (without arrest)  ▢ Searched for condoms  ▢ Other property taken (without arrest)  ▢ Propositioned to exchange sex by:  undercover/plain clothes cop and/or;  Cop on duty and/or;  Cop off duty  ▢ Coerced into providing sexual favours (e.g. in exchange for freedom from arrest) by  undercover/plain clothes cop and/or;  Cop on duty and/or;  Cop off duty | For each option   - Any in the last 6month   And/or   - While working (sex work, L6M) | - Yes (‘While working (sex work, L6M)’ to any of ‘Told to move on’, ‘Police raid’, ‘Threatened with arrest/detainment/fine’, ‘Searched’, ‘Followed’, ‘Picked up and driven elsewhere to work’, ‘Verbally harrassed’, ‘Parked nearby/drove by repeatedly’, ‘Detained’, ‘Physically assaulted’, ‘Drugs/drug use equipment taken’, ‘Condoms taken’, ‘Searched for condoms’, ‘Other property taken’, ‘Propositioned to exchange sex’, ‘Coerced into providing sexual favours’)   vs   - No (None of the above) and/or N/A (no sex work in the L6M) |
| Confounder variables | | | |
| Age | How old are you? | - Age in years | Continuous variable (years). Time updated at follow-up based on age at baseline and interview date |
| Minority sexual orientation | Which of the following best  describes your sexual orientation (check all that apply):  ▢ Straight  ▢ Gay  ▢ Lesbian  ▢ Bisexual  ▢ Asexual  ▢ Queer  ▢ Other (specify:______________)  First Nations, Métis or Inuit participants:  ▢ Two spirited | Check each option that applies | - Sexual minority (Any of ‘Gay’, ‘Lesbian’, ‘Bisexual’, ‘Two spirit’, ‘Asexual’, ‘Queer’, ‘Other’)   vs   - Straight (‘Straight’ ONLY) |
| Gender minority | Which of the following best describes  your gender identity (check all that apply):  ▢ Transgender  ▢ Cisgender  ▢ Intersex  ▢ Transexual  ▢ Genderqueer  ▢ Other (specify:______________)  First Nations, Métis or Inuit participants:  ▢ Two spirited | Check each option that applies | - Gender minority (Any of ‘Transgender’, ‘Intersex’, ‘Transexual’, ‘Two spirit’, ‘Genderqueer’, ‘Other’)   vs   - Cisgender (‘Cisgender’ ONLY) |
| Racialization | What ethnicity(s) do you identify with? (Ethnic origin or ancestry refers to the ethnic or cultural origin of a person’s  ancestors)  ▢ White  ▢ Chinese  ▢ Taiwanese  ▢ Indian (South Asian)  ▢ Pakistani  ▢ Bangladeshi  ▢ Vietnamese  ▢ Korean  ▢ Japanese  ▢ Filipina  ▢ Thai  ▢ Sri Lankan  ▢ Latin, Central or South American (specify:______)  ▢ Middle Eastern (specify:_____________)  ▢ African (specify:______)  ▢ Black (specify:_______)  ▢ First Nations / Aboriginal | For each option check   - Any   And/or   - Most | - Indigenous (Any of ‘First Nations’, ‘Metis’, ‘Inuit’)   vs   - Other racialized person (Black/ Person of Colour) (Any of ‘Chinese/Taiwanese’, ‘Vietnamese’, ‘Korean’, ‘Japanese’, ‘Thai’, ‘Filipina’, ‘Indian’, ‘Pakistani’, ‘Bangledeshi’. ‘Sri Lankan’, ‘Latin American’, ‘Middle Eastern’, ‘African’, ‘Black’, relevant ‘Other’)   vs   - white (‘white’ ONLY) |
| Mental health diagnosis | Have you been diagnosed  with this condition?  ▢ Depression  ▢ Anxiety  ▢ Post-traumatic stress disorder  ▢ Schizophrenia/schizoaffective  ▢ Bipolar disorder  ▢ Psychosis (drug-induced)  ▢ None | For each option check   - Ever   And/or   - In the last six months | - Yes (‘Ever’ to ‘Diagnosed with this condition?’ for any condition listed)   vs   - No (None of the above) |
| Alcohol use | Which non-injection drugs have you used?  ▢ Beer/cooler/wine  ▢ Liquor  ▢ Cooking wine/ rubbing alcohol/ mouthwash  ▢ Other alcohol | For each option:   - Yes   vs   - No   AND  How often? | - Daily (‘Daily’ to any of ‘Beer/cooler/wine’, ‘Liquor’, ‘Cooking wine/rubbing alcohol/mouthwash’, ‘Other alcohol’)   vs   - Less than daily (‘Yes’ to alcohol_l6m but not ‘Daily’ to any)   vs   - None (‘No’ to alcohol_l6m) |
| Injection drug use | In the last 6 months, have you ever used a needle to fix or muscle (even once) [excluding prescribed injectionable opiate  or stimulant therapy]? | - Yes   vs   - No | - Yes (‘Yes’ to ‘In the last 6 months’ for any injection drugs)   vs   - No (None of the above) |
| Nonfatal overdose | In the last 6 months, have you overdosed by accident  (i.e. where you’ve had a negative reaction by using too much  drugs)? | - Yes   vs   - No | - Yes   vs   - No |
| Hospitalization | In the last 6 months, have you been admitted to the  hospital/ stayed overnight? | - Yes   vs   - No | - Yes   vs   - No |

*From AESHA questionnaire

**Please note there are multiple updated/adapted versions of the baseline and follow-up questionnaires from the over 27 follow-up periods. The questions listed here remained stable over this specific study period.
